# The Impact of Sterile Instrument Set Wrapping Defects on Trauma and Orthopaedic Surgery Theatre Lists

**DOI:** 10.7759/cureus.29861

**Published:** 2022-10-03

**Authors:** Fitzgerald Anazor, Vusumuzi Sibanda, Kalsoom Altaf, Lisa Downer, Jai Relwani

**Affiliations:** 1 Trauma and Orthopaedics, William Harvey Hospital, Ashford, GBR; 2 Decontamination Contract Support Officer, Kent and Canterbury Hospital, Canterbury, GBR

**Keywords:** trauma and orthopaedics, orthopaedic implant-related infection, financial impact, sterile instrument wrappings, infection control measures, surgical equipment

## Abstract

Introduction

Surgical site infections (SSIs) are a universally dreaded complication of any surgical procedure. The goal of this single-center study was to examine the issue of orthopaedic instrument wrapping defects with a focus on the importance of a high level of surveillance to enable identification of these defects in order to reduce the risk of instrument contamination. We also evaluated the impact on patient care, theatre staff, hospital finances and resource utilization during a defined study period in order to stimulate critical discussion and further research into potentially eliminating this problem via change in practice and advances in technology. To the best of our knowledge, this will be the first paper that looks at this problem from the above perspective within the United Kingdom National Health Service.

Methods

We conducted a prospective service evaluation project over a 30-week period from December 2021 to July 2022 across our three hospital sites within the United Kingdom National Health Service. We collated data on defects found in orthopaedic instrument wrappings as detected by visual inspection under ambient or theater lighting and the resulting surgical case cancellations. Defect types included in the study were all puncture holes, abrasions or tears visible to the naked eye irrespective of their size.

Results

A total of 601 orthopaedic sets were rejected during the study period due to defects identified in the sterile instrument wrappings. Of these, 437 were due to holes/tears in the wrapping, 129 were due to wet inner wrappings and 35 were due to insecure wrappings. This directly resulted in same-day cancellation of 13 surgical cases or 0.27% of booked cases with extra sets required for the other affected cases. These 13 cases could not proceed as they involved loan kits where no remedial action could be taken. Remedial action was required for the other 588 operation list cases affected by the sterile wrapping defects. The majority of the identified defects and resulting theatre case cancellations (61.5%) were in hip and knee arthroplasty surgery. The calculated potential financial loss due to these problems was £145,000 over the seven-month study period. This financial cost is equivalent to the best practice top-up tariff in England for treating approximately 108 hip fracture patients based on £1,335 per patient.

Conclusion

Our study identified defects in the sterile instrument wrappings affecting both the inner with or without involving the outer wrapping layer and resulting in cancellation of elective and trauma orthopaedic cases with resultant clinical and financial implications. There is a need to be more vigilant in identifying defects in drapes. Further research is warranted to improve ways of identifying defects in sterile wrappings and devise new protective mechanisms during sterilisation that can eliminate the use of sterile instrument wrappings.

## Introduction

Surgical site infection is a potentially catastrophic problem in surgery and the sequelae are especially devastating in orthopaedic surgery due to the use of prostheses and implants. This can lead to prolonged patient hospital stay, reduced quality of life, increased costs to the health service, lower patient-reported outcome measures, more time spent in medical care delivery per patient, increased readmission/reoperation rates, potential limb loss and even mortality in severe cases [1-3]. 

The risk of surgical site infection in orthopaedic surgery ranges from 0.1-1% for non-implant surgery to 1-2% for primary arthroplasty surgery and up to 3-10% for revision hip/knee surgery [4-6]. Reducing surgical site infection is a major goal in orthopaedic surgery. Prevention methods include meticulous attention to aseptic techniques and avoidance of breach in surgical instrument sterility. Surgical instruments are typically arranged in instrument trays in a way that facilitates efficient utilization of instruments during a surgical operation. These instrument trays are usually wrapped in special materials that are strong enough to withstand autoclaving yet porous enough to allow for adequate steam sterilization in the autoclave. 

Prior to their use, surgical instrument trays are assessed for any breaches in the wrapping or packaging; including tears, holes, wet wrapping, abrasions as well as poorly secured wrapping. Breaches can occur prior to sterilization due to ineffective packaging, during sterilization or after sterilization, especially during storage or transportation. Upon identification of a breach or defect, the instrument tray with its contents is assumed to be unsterile, re-wrapped and sent back for repeat sterilization. This can lead to significant delays which can have attendant negative impact on patient care as well as staff morale, theatre time and resource utilization. There are also increased potential financial costs arising from delays and cancellations to same-day theatre lists. 

The coronavirus disease 2019 (COVID-19) pandemic has worsened the National Health Service (NHS) United Kingdom (UK) trauma and orthopaedic surgery waiting list which as of March 2022, stands at 730,000 in England [7]. To speed up clearance of this backlog, an efficient utilization of surgical theatre time and resources is of paramount importance. Also, emergency departments are under significant pressures and cancelled or delayed trauma surgical cases can potentially lead to deterioration of patients’ health, prolonged hospital admission for the patient and potentially add to the already stretched pressures on hospital staff and resources. 

The questions this study aims to address include: 

1. What proportion of orthopaedic sterile instrument tray wrappings have visibly identifiable wrapping defects? 

2. What percentage of orthopaedic theatre list surgical cases are cancelled or postponed on the day of surgery due to these defects identified in the sterile instrument tray wrappings? 

3. What are the potential direct financial costs to the healthcare system arising from these list cancellations, postponements, resultant prolonged hospital stay for patients and theatre-time utilization inefficiency? 

This single-center study examined the issue of orthopaedic instrument wrapping defects with a focus on the importance of a high level of surveillance to enable identification of these defects in order to reduce the risk of instrument contamination. We also evaluated the burden on patient care, theatre staff, hospital finances and resource utilization during a defined study period in order to stimulate critical discussion and further research into potentially eliminating this problem via change in practice and advances in technology. To the best of our knowledge, this will be the first paper that looks at this problem from the above perspective within the United Kingdom National Health Service. 

## Materials and methods

We conducted a service evaluation project, collating data for defects in orthopaedic instrument wrappings resulting in surgical case cancellations or delays over a 30-week period between December 6, 2021 and July 1, 2022. We included both elective and trauma list cases. The study was conducted across three hospital sites within the East Kent Hospitals University NHS Foundation Trust in the United Kingdom. The study was registered as a service evaluation project with our hospital's clinical audit team. Data was collated using the Excel sheet application (Microsoft, Redmond, WA, USA). 

In our unit, orthopaedic instrument trays are covered with Alcoban^TM^ wrapping (Elis Surgical Solutions, Wednesbury, UK) or H400^TM^ wrapping (Halyard, Alpharetta, GA, USA) prior to undergoing autoclaving. The Alcoban wrapping is made of 99% polyester microfiber and 1% carbon fiber. Its microporous construction is designed to allow for sterilization and the material strength is theoretically more resistant to abrasion and tears. Comparatively, the H400 wrapping is made up of 97% polypropylene, 2% phthalocyanine blue pigment, approximately 1% titanium oxide and 0.009% titanium oxide anti-static treatment. These specialized wraps allow for instrument tray/set opening using an aseptic technique. The sterile wrap models above are suitable for moderate to heavy instrument sets weighing up to a maximum of 11-12 kg. The outer layer of wrapping is usually blue or green with an inner white layer for easier defect identification. 

Sterile instrument trays or containers with the wrapping in situ are usually inspected visually for holes in either the inner or outer wrapping by theatre nurses who are trained and have the requisite skills to perform this. The visual inspection is usually carried out under ambient and/or theatre lighting. Tears involving the inner layer of wrapping imply a potential breach of the sterile instrument tray. In such scenarios, such trays were deemed to be contaminated and were rejected for the surgical case.

**Figure 1:**
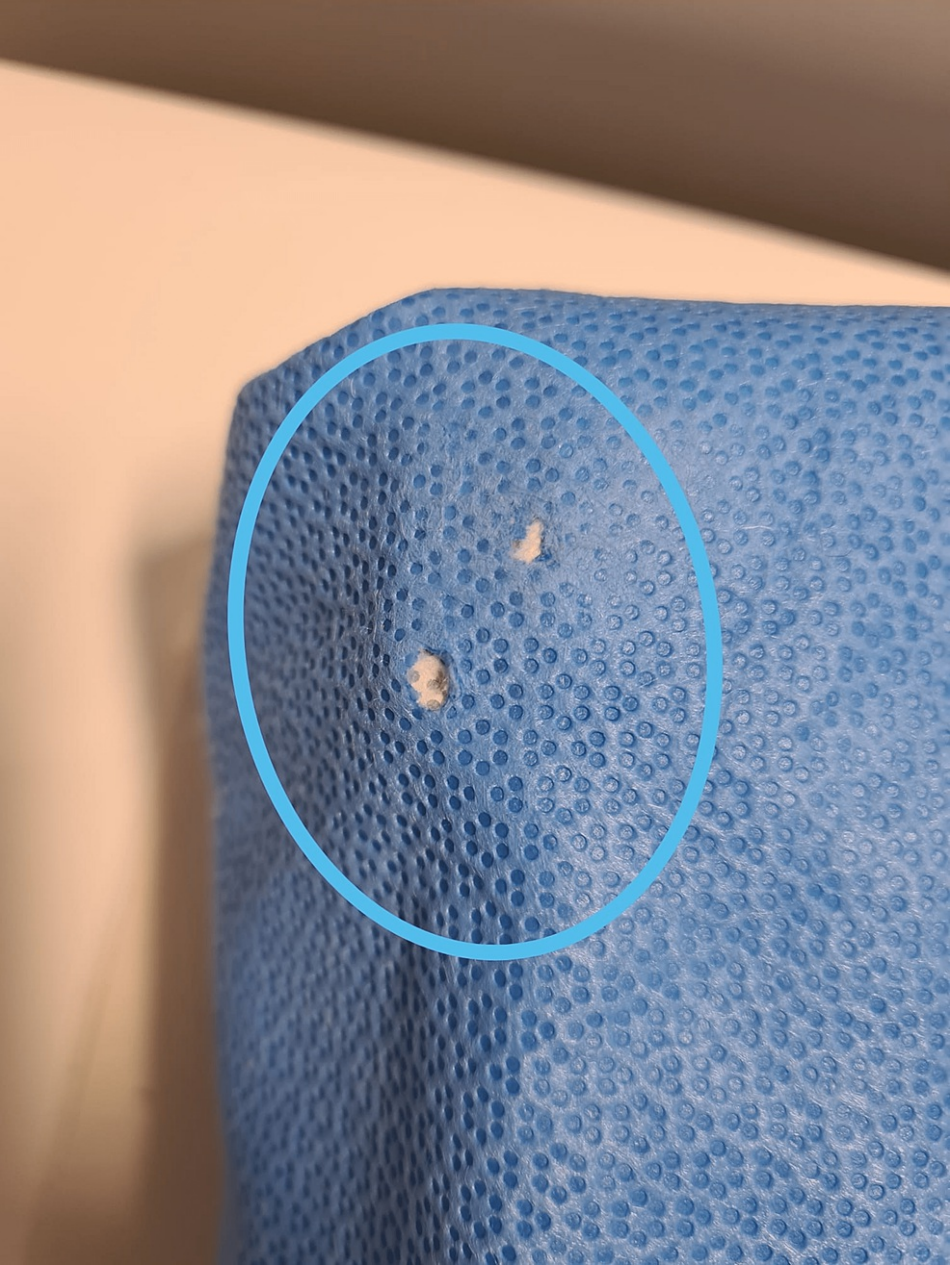
An example of two circular holes found in one of the sterile instrument wrappings which are relatively easier to identify via visual inspection

**Figure 2:**
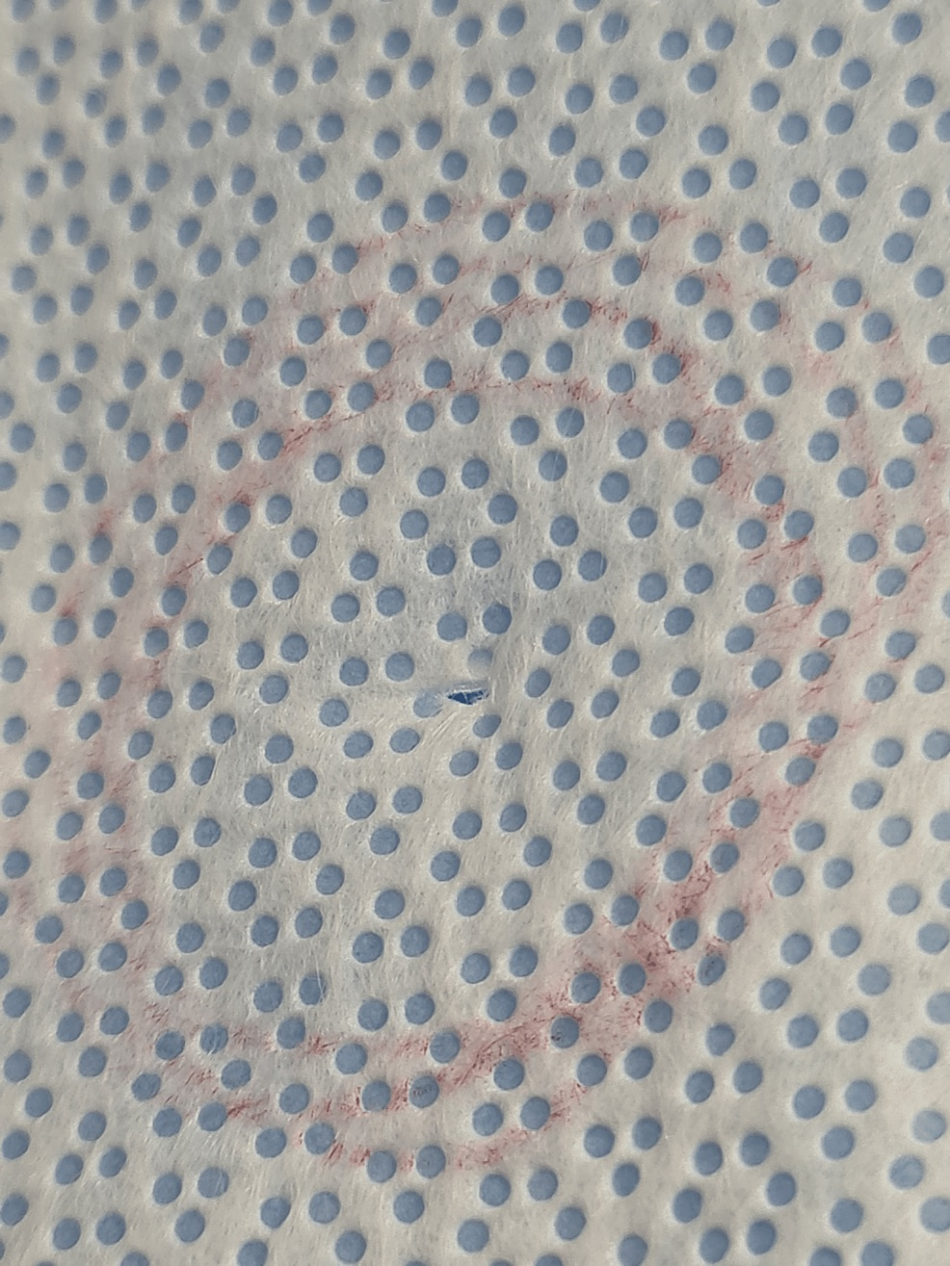
A small puncture hole measuring <2.5 mm in diameter and may be easily missed via visual inspection

**Figure 3:**
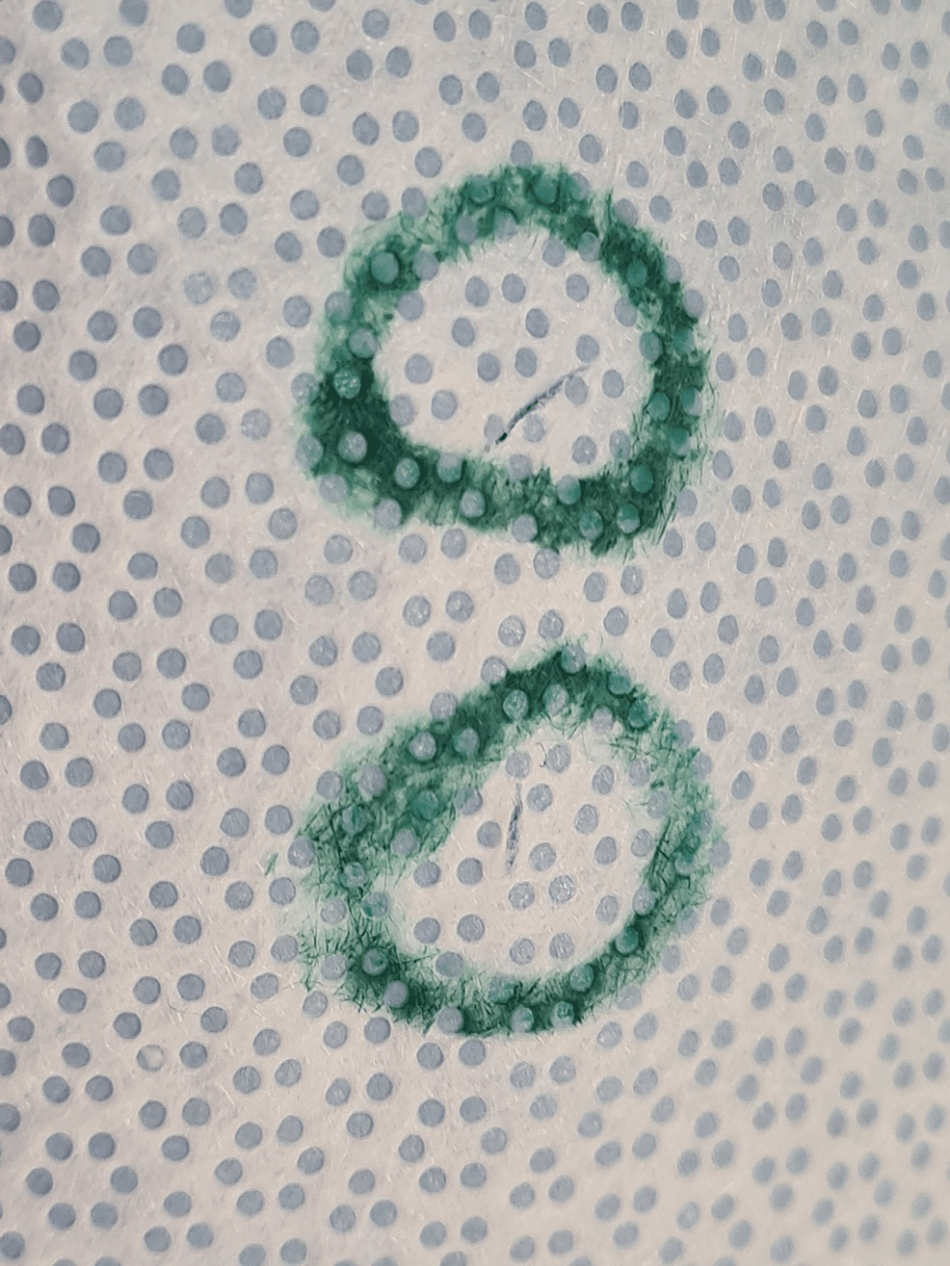
Two small linear tears >2.5 mm in length identified in another sterile tray wrapping located in the central third of an instrument tray

**Figure 4:**
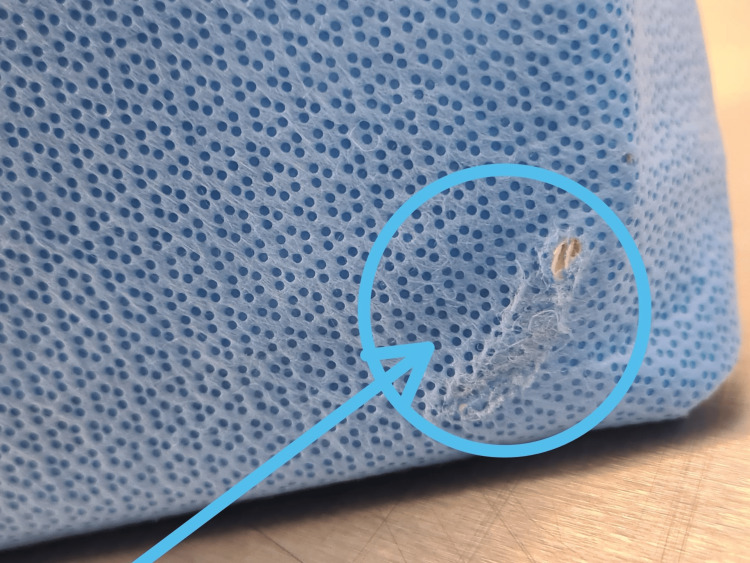
Ragged abrasive tear identified in one of the sterile tray wrappings, located at the corner of the instrument tray

Defect types included in the study were all puncture holes, abrasions or tears visible to the naked eye irrespective of their size. We also included data for wet and insecure wrappings resulting in instrument set rejection. Holes can be penetrating in an inside-out or outside-in pattern and are usually caused by sharp instruments. Shear or abrasion-type tears are due to heavy instrument sets being dragged on a surface during transport or handling. Pressure defects causing compression of the wrapping usually affect the instrument tray corners and occur mainly during transport. Generic tears occur from other miscellaneous reasons during handling (Figures 1-4).

For our study, a surgical case cancellation was defined as an unplanned same-day deferral or termination of an elective or trauma case prior to its commencement due to sterile instrument wrapping defects identified on the instrument tray.

## Results

Analysing the results for the 601 rejected orthopaedic instrument sets during the 30-week study period, 437 were due to holes/tears in the sterile instrument wrappings, 129 were due to wet instrument tray wrappings and 35 were due to insecure wrapping. The rejection of the 601 orthopaedic instrument sets resulted in a total of 13 procedure cancellations as a result of defects identified in the inner and outer layers of the sterile instrument set wrapping during the study period (Table 1). These 13 cases could not proceed as they involved loan kits where no remedial action could be taken. Remedial action for the other 588 operation list cases affected by the sterile wrapping defects included use of alternative extra instrument trays when available, change of list order, photography of the defects, incident reporting and return of the rejected instrument tray for reprocessing. Change of list order was not feasible if an affected case was scheduled as the last case of the day. 

**Table 1:** Summary of the sterile wrapping defects in relation to Orthopaedic surgery subspecialty category and number of surgical case cancellations

ORTHOPEDIC SUB-SPECIALTY OR TYPE OF SURGERY AFFECTED	NUMBER OF KITS WITH WRAPPING DEFECTS	NUMBER OF RESULTING SURGICAL CASE CANCELLATIONS
Hip arthroplasty	107	4
Hip arthroscopy	1	1
Knee arthroplasty	135	2
Knee/shoulder arthroscopy	8	1
Other major hip/femur surgery	9	0
Other major knee surgery	28	0
Shoulder arthroplasty	22	0
Elbow surgery	1	1
Hand and wrist surgery	3	0
Spine surgery	5	0
Foot and ankle surgery	7	1
Power tools and other general miscellaneous instrument trays including related anesthesia kits	275	3

Defects in the sterile instrument tray wrappings affected a wide variety of orthopaedic surgical instruments as summarised in Table 1, with the majority being hip and knee arthroplasty surgery sets. The total number of booked Trauma and Orthopaedic surgical operation cases during the same period was 4752. Comparing this with the 13 cancellations during the study period, this means that approximately 0.27% of operation list surgical cases were cancelled due to sterile instrument wrapping defects. 

The potential financial cost from loss of tariffs from the 13 cancelled cases during the 30-week period in our study amounts to £80,000 based on the 2022/2023 NHS tariff workbook that details the tariff prices for the different orthopaedic procedures [8]. In addition, the cost of sterilisation per orthopaedic instrument tray ranges from £35-£42 [9,10], equating to approximately £25,000 for the 601 sterile wrapping defects sent back for repackaging and re-sterilisation. Furthermore, the opportunity and capacity costs per minute of lost theatre utilisation time is approximately £25 [11], equating to approximately £40,000 for the 13 cancelled cases, assuming a 120-minute allocated theatre time per case. Thus, the total potential costs resulting from the above-identified issues is £145,000 over the 30-week period.

## Discussion

Surgical site infections in orthopaedic surgery can result from post-sterilisation contamination of surgical instruments [12]. Holes and other breaches in surgical tray wrappings including puncture holes as small as 1.1 mm in diameter can lead to instrument tray contamination [13]. This highlights the importance of inspecting sterile instrument tray/kit wrappings for defects prior to opening the contents for use. 

The most commonly used method for identifying these defects in our setting and in many other institutions is through visual inspection under ambient or theatre lighting, generally by the scrub nurse prior to opening the instrument tray [14]. Due to the variability in defect size, the sensitivity of visual inspection for identifying these holes varies from 7% for defects <2 mm in diameter to as high as 87% for defects >2.4 mm in diameter [15]. 

Same-day elective surgery cancellation has deleterious effects on the patient, including negative emotional impact, increased financial burden and potential health deterioration [6,16]. Delays in performing urgent trauma surgery or use of second-line surgical implants due to inability to use instrument kits as a result of defective sterile wrapping can affect surgical outcomes. Financial implications to the hospital can arise from loss of potential earnings from cancelled cases, increased cost of re-packaging/re-sterilization of rejected instrument kits and loss of any applicable performance tariffs. Staff morale can also be affected due to theatre delays, need to reschedule cancelled cases on future theatre list slots within 28 days and repeat inspections/repackaging of instrument sets. 

From the results, the potential financial costs arising from these sterile instrument wrapping defects are not insignificant at £145,000 over a seven-month period. Notably, this sum excludes the potential costs arising from possible prolonged in-patient stay and costs arising from hospitals needing extra instrument kits for possible remedial action. This financial cost is equivalent to the best practice top-up tariff for treating approximately 108 hip fracture patients based on £1,335 per patient [17]. This highlights an area of potential cost savings. Although eliminating wrapping defects completely may be an ambitious challenge currently, identifying the presence of these earlier, such as the day before surgery, can minimise the incidence of this problem. However, sets need to be opened to identify holes in the inner wrapping and hence, this solution might be impractical until the last minute. 

Looking at this problem from a different perspective, having a higher detection rate for sterile instrument set wrapping defects may reflect the thoroughness of the inspection being conducted by the theatre staff. Therefore, it is important that hospital theatres with relatively low incidence of defective surgical instrument wrappings, maintain or even reassess their surveillance methods to ensure they are not missing this potentially serious source of instrument contamination. 

Our study also highlights the fact that wrapping defects can potentially affect most types of instrument trays/sets. The heavier sets used in hip and knee arthroplasty were affected the most. A balance between durability of the wrapping and porosity to allow for effective sterilization via autoclaving is the goal of current methods. Careful handling, minimizing cross-site kit transportation, appropriate storage of instruments, proper wrapping techniques and use of closed rather than open trays are potential ways of reducing post-sterilization wrapping defects. An example of a relatively new method is the use of sterilization pods that avoids the use of instrument tray wrappings, thus eliminating this problem and potentially avoiding the aforementioned theatre list burden [18]. We hope that further research leading to technological advancements will provide new cost-effective and efficient ways of protecting orthopaedic instruments during and after sterilisation. 

We acknowledge some of the limitations of this study. Firstly, not all sterile instrument wrapping defects can be detected via visual inspection. In addition, there is also a potential for inter- and intra-observer variations in detecting these defects. Finally, we have not measured the sizes of all the identified defects as our focus was mainly on highlighting the importance of effective surveillance in identifying this problem through the standard operating procedures in-place and assessing the resultant burden on the efficiency of the orthopaedic surgical list. 

## Conclusions

Surgical site infections are a dreaded complication of any surgical procedure, with potentially damaging implications to both the patient and the treating clinician. Steps must be taken at each stage of the patient's operative journey to minimise the risk of developing SSIs by early identification and remediation of any breach in peri-operative asepsis or sterility.

Our study examined the problem of routinely identified defects in the sterile instrument wrappings affecting the inner with or without the outer layer and resulting in cancellation of elective and trauma orthopaedic cases with resultant clinical and financial implications. There is a need to be more vigilant in identifying defects in these sterile instrument wrappings. In addition, reducing this problem can lead to cost savings especially in global healthcare settings and potentially contribute to green surgical initiatives through reduced material waste and energy expenditure from repackaging and re-sterilisation. Further research is warranted to improve ways of identifying defects in sterile wrappings and devise new protective mechanisms during sterilization that can eliminate the use of sterile instrument wrappings. 
